# 369. Alternative Workflow for SARS-CoV-2 Testing Using a Heat Lysis Protocol for Respiratory Specimens

**DOI:** 10.1093/ofid/ofab466.570

**Published:** 2021-12-04

**Authors:** Meghna Yadav, Tiffany Martinez, Isabel Regoli, Osvaldo Hernandez, Phuong Le, Heather Carolan, Brad Brown, Karen Menge

**Affiliations:** 1 ChromaCode, Carlsbad, California; 2 ChromaCode Inc, Carlsbad, California; 3 ChromaCode Inc., Carlsbad, California

## Abstract

**Background:**

The SARS-CoV-2 pandemic has demonstrated the need for streamlined workflows in high-throughput testing. In extraction-based testing, limited extraction reagents and required proprietary instrumentation may pose a bottleneck for labs. As a solution, ChromaCode developed a Direct Extraction protocol for the HDPCR™ SARS-CoV-2 Assay, distributed in accordance with the guidance on Policy for Coronavirus Disease-2019 Tests During the Public Health Emergency, Section IV.C., which allows for the processing of specimens without an extraction system. In lieu of an extraction system, the Direct Extraction protocol uses a thermal cycler to lyse and inactivate specimens which are directly added to the Polymerase Chain Reaction (PCR).

**Methods:**

The Limit of Detection (LoD), Clinical Performance, and effect of Interfering Substances was determined for the Direct Extraction protocol. The LoD was established on 6 PCR platforms with dilutions of inactivated SARS-CoV-2 virus spiked into residual, negative nasopharyngeal swab (NPS) matrix. Clinical performance was assessed with 48 positive and 50 negative frozen retrospective samples using the Direct Extraction protocol compared to an external Emergency Use Authorized (EUA) comparator assays (cobas® Liat® SARS-CoV-2 & Influenza A/B assay and the Hologic Panther Fusion® SARS-CoV-2 Assay respectively) on three PCR platforms. The Direct Extraction protocol was evaluated for performance in the presence of 13 potentially interfering substances that can be present in a respiratory specimen.

**Results:**

The LoD of the Direct Extraction protocol ranges from 1000 – 3000 genomic equivalents (GE)/mL. The clinical performance of the assay was 95.8% positive agreement (95% CI of 84.6% - 99.3%) and 100% negative agreement (95% CI of 90.9% - 100% or 91.1% – 100%) across all three PCR platforms tested. The viral target was detected at 3X LoD for all interferents tested.

**Conclusion:**

The Direct Extraction protocol of ChromaCode’s SARS-CoV-2 Assay is a sensitive test that eliminates the need for sample extraction and performs very well against traditional extraction-based workflows. The inclusion of this protocol can reduce costs, reliance on extraction systems, and time associated with extraction-based protocols.

**Disclosures:**

**Meghna Yadav, Ph.D. Molecular Biology**, **ChromaCode Inc.** (Employee, Shareholder) **Tiffany Martinez, n/a**, **ChromaCode** (Employee, Shareholder) **Isabel Regoli, MS, Bioinformatics**, **ChromaCode** (Employee, Shareholder) **Osvaldo Hernandez, B.S., Molecular Biology**, **ChromaCode** (Employee, Shareholder) **Phuong Le, B.S., Biochemistry**, **ChromaCode** (Employee, Shareholder) **Heather Carolan, Masters, Computational Molecular Biology**, **ChromaCode** (Employee, Shareholder) **Brad Brown, Ph.D Biomedical Sciences**, **ChromaCode** (Employee, Shareholder) **Karen Menge, Ph.D. Biochemistry**, **ChromaCode** (Employee, Shareholder)**ChromaCode** (Employee, Shareholder)

